# Knocking down of heat-shock protein 27 directs differentiation of functional glutamatergic neurons from placenta-derived multipotent cells

**DOI:** 10.1038/srep30314

**Published:** 2016-07-22

**Authors:** Yu-Che Cheng, Chi-Jung Huang, Yih-Jing Lee, Lu-Tai Tien, Wei-Chi Ku, Raymond Chien, Fa-Kung Lee, Chih-Cheng Chien

**Affiliations:** 1 Department of Medical Research, Cathay General Hospital, Taipei, Taiwan; 2 Institute of Biomedical Engineering, National Central University, Jhongli, Taiwan; 3 Center for Biocellular Engineering, National Central University, Jhongli, Taiwan; 4 School of Medicine, Fu Jen Catholic University, New Taipei City, Taiwan; 5 Department of Biochemistry, National Defense Medical Center, Taipei, Taiwan; 6 Department of Respiratory Therapy, Fu Jen Catholic University, New Taipei City, Taiwan; 7 Department of Obstetrics and Gynecology, Cathay General Hospital, Taipei, Taiwan; 8 Department of Anesthesiology, Cathay General Hospital, Taipei, Taiwan

## Abstract

This study presents human placenta-derived multipotent cells (PDMCs) as a source from which functional glutamatergic neurons can be derived. We found that the small heat-shock protein 27 (HSP27) was downregulated during the neuronal differentiation process. The *in vivo* temporal and spatial profiles of HSP27 expression were determined and showed inverted distributions with neuronal proteins during mouse embryonic development. Overexpression of HSP27 in stem cells led to the arrest of neuronal differentiation; however, the knockdown of HSP27 yielded a substantially enhanced ability of PDMCs to differentiate into neurons. These neurons formed synaptic networks and showed positive staining for multiple neuronal markers. Additionally, cellular phenomena including the absence of apoptosis and rare proliferation in HSP27-silenced PDMCs, combined with molecular events such as cleaved caspase-3 and the loss of stemness with cleaved Nanog, indicated that HSP27 is located upstream of neuronal differentiation and constrains that process. Furthermore, the induced neurons showed increasing intracellular calcium concentrations upon glutamate treatment. These differentiated cells co-expressed the N-methyl-D-aspartate receptor, vesicular glutamate transporter, and synaptosomal-associated protein 25 but did not show expression of tyrosine hydroxylase, choline acetyltransferase or glutamate decarboxylase 67. Therefore, we concluded that HSP27-silenced PDMCs differentiated into neurons possessing the characteristics of functional glutamatergic neurons.

Placenta-derived multipotent cells (PDMCs) are a population of multipotent cells that can be isolated from the human placenta. Unlike embryonic stem cells, PDMCs are a stem-cell source that can be collected without ethical problems. These multipotent cells have the ability to differentiate into osteoblasts, adipocytes, and hepatocytes^1^,^2^ and exhibit many markers that are common to mesenchymal stem cells; however, unlike other cell sources, no intrusive procedure is necessary to obtain PDMCs. Several applications of mesenchymal stem cells have been proposed, such as treatment for liver disease and suppressed proinflammatory cytokine function, which implies that PDMCs may have the same translational applications without raising the technical and safety concerns of other stem cells^3^,^4^,^5^.

Neural tissue is thought to have a limited capacity for repair after injury, and cellular grafts generated from stem cells may provide replacement therapies that are consistent and reproducible^5^. Therefore, the broad spectrum of mechanisms that underlie neuronal differentiation must be identified. Hippocampal progenitor cells, bone-marrow mesenchymal stem cells, and PDMCs have been proved to have the ability to initiate neural differentiation^6^,^7^. During this process, cells exhibit morphological changes including neurite outgrowth and the expression of various neuron-specific molecules^7^. However, the mechanisms underlying the differentiation of stem cells into specific cell lineages are complicated and mostly unknown^8^,^9^.

To understand the protein expression profiles during neuronal differentiation, we used proteomics approaches to generate a panel of upregulated and downregulated proteins that were involved in the neuronal differentiation of PDMCs^10^. In this study, we found that a protein identified by proteomic approaches as the small heat-shock protein 27 (HSP27) was downregulated, and we verified this downregulation by assessing its transcriptional and translational levels during neuronal differentiation.

The number and distribution of excitatory glutamatergic and inhibitory gamma-aminobutyric acid (GABAergic) neurons in the human central nervous system must be precisely controlled. The imbalance or loss of glutamatergic and GABAergic neurons is observed in many nervous system disorders, such as Alzheimer’s disease^11^, age-related macular degeneration^12^ and mental retardation^13^. Although Alzheimer’s disease affects many types of neurons, including non-glutamatergic interneurons and cholinergic projection neurons, as well as glia, it is still desirable to understand the molecular mechanisms that govern glutamatergic neuron differentiation and to develop a consistent and reproducible glutamatergic neuron source for elucidating the aetiology of neurological disorders.

Heat-shock proteins (HSPs) are classified into five families according to their molecular size^14^. Their expression can be induced by different types of stress and specific cellular stages, such as development, differentiation, and tumourigenesis^15^. HSPs show strong cytoprotective effects and behave as molecular chaperones for other cellular proteins^16^. The differential expression levels of HSPs have been reported to be involved in neural differentiation and certain neurodegenerative disorders^17^,^18^,^19^. Among the HSPs, HSP27 is well known for its clinical significance in neuropathy^20^,^21^. In the current study, we demonstrated that HSP27 plays a role distinct from its classical heat-shock response and that instead contributes to orchestrating neuronal differentiation. Overexpression of HSP27 disrupted neuronal differentiation, and knockdown of HSP27 enhanced the induced neuronal differentiation process of PDMCs. Other evidence, including immunostaining for the expression pattern of HSP27 during mouse embryonic development and immunoblotting for several apoptosis and stem cell markers, suggests that HSP27 is physiologically crucial during neuronal differentiation.

## Results

### Neuron-like morphology, Neuron specific enolase (NSE) signals, and downregulation of HSP27 expression in IBMX-treated PDMCs

**Figure 1:**
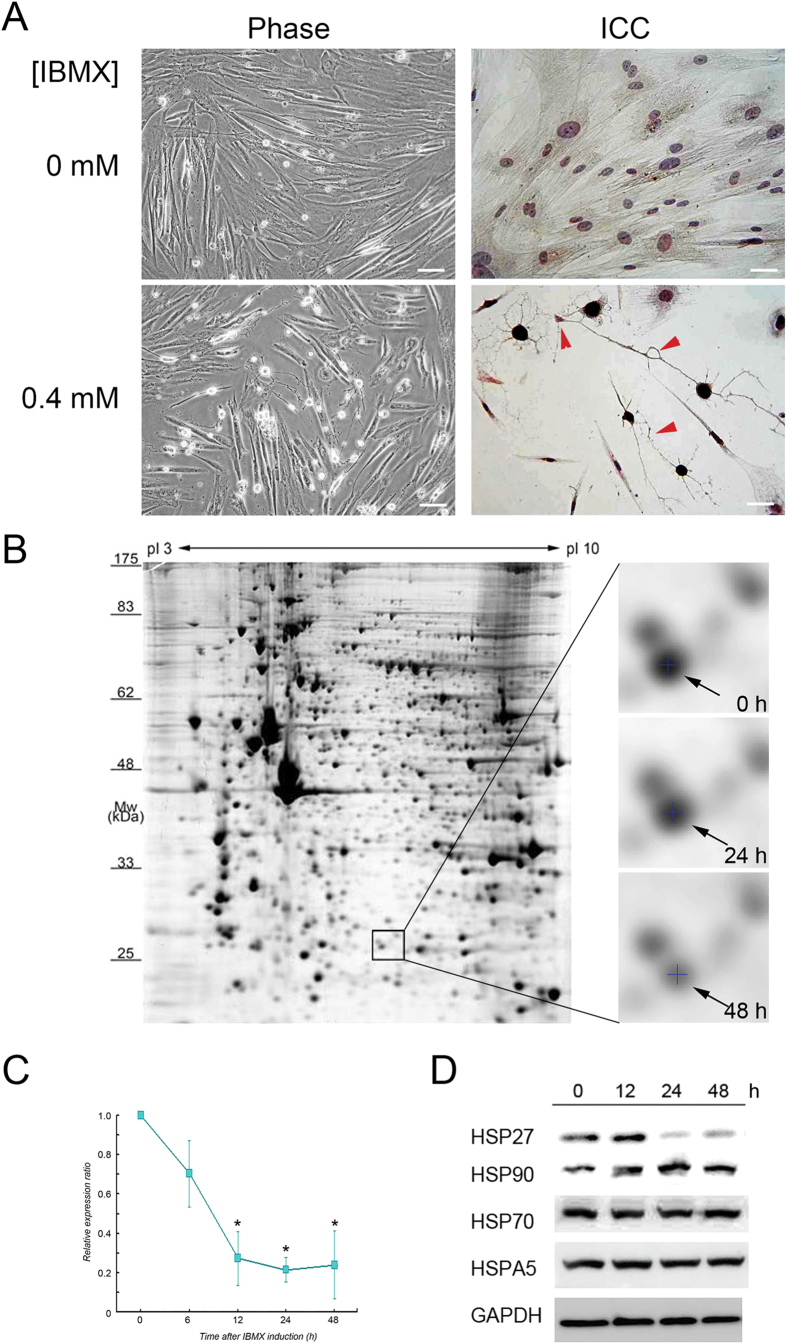
Cell model and expression profiles of HSP27 in neurons differentiated from PDMCs. (**A**) Cell morphology showing the differentiation of neurons derived from PDMCs. Phase contrast images of PDMCs (left upper panel) and neuron-like cells induced by IBMX (left lower panel). Immunocytochemistry analysis of PDMCs (right upper panel) and differentiated neuron-like cells using anti-NSE antibody (right lower panel). Induced neurons extended neurites and formed synaptic connections with each other (red arrowheads). Scale bar = 100 μm. (**B**) Temporal expression of HSP27 protein in the study model was profiled using 2D-PAGE. PDMCs were harvested at the indicated time points after treatment with IBMX. The proteins at each time point were profiled using 2-DE-PAGE. Cell extracts (300 μg) from each time point were separately subjected to the 2-DE procedure, and protein spots were visualized with silver staining. The rectangle represents the location of HSP27 in 2D-PAGE images. The HSP27 expression level in each 2-DE-PAGE was selected, marked by a cross and indicated with arrows, showing that the protein expression of HSP27 at different time points decreased after IBMX induction (right panel). (**C**) qPCR analysis of mRNA expression in the PDMCs after IBMX induction. Each time point was evaluated in five independent experiments. The star indicates statistical significance with *p* value < 0.05. (**D**) Immunoblots for HSP27, HSP90, HSP70 and HSPA5 after IBMX induction of PDMCs. Cell extracts (30 μg) were prepared from induced PDMCs at the indicated time points (0, 12, 24 and 48 h). GAPDH was used as an internal control.

Cultured cells were identified as PDMCs using a flow cytometric approach that examined the expression of surface markers, as previously reported^7^. Two days after induction with 0.4 mM IBMX, the morphology of some of these cells had transformed from a spindle shape into neuron-like cells with fibre outgrowth. As shown in Fig. 1A, these neuron-like cells presented a strong immunocytochemical signal for the anti-NSE antibody compared with the panel obtained from uninduced cells, and some connecting synaptic terminals were also observed in the IBMX-induced cells.

To characterize the proteomics profile changes that occurred during the process of the IBMX-induced differentiation of PDMCs into neurons, lysates of PDMCs were analysed using 2-dimensional electrophoresis (Fig. 1B). A spot with a pI value of approximately 6 and a molecular weight of approximately 27 kDa showed reduced intensity at various incubation times after IBMX induction (Fig. 1B, right panel) and was identified as HSP27 in a tandem-MS analysis (Table 1). The expression level of the HSP27 mRNA in PDMCs at various time points after IBMX induction was also quantified using qPCR with HSP27-specific primers (Fig. 1C). Compared to the levels of HSP27 mRNA detected in cells without additional incubation (0 h), a significant downregulation of the HSP27 mRNA (*P* = 0.0007) was observed in cells at 6 h of incubation after IBMX induction. The lowest levels of HSP27 mRNA expression were detected in cells that underwent longer incubation with IBMX (12, 24, and 48 h) (*P* < 0.0001). On average, qRT-PCR analysis revealed that approximately 20% of the original HSP27 mRNA was retained after 12 h of incubation after IBMX induction. However, no significant changes (*P* > 0.05) in the expression level of HSP27 mRNA were found during longer incubation periods (12 to 48 h). The gradual downregulation of HSP27 protein was further confirmed by immunoblot analysis using an anti-HSP27 antibody, whereas the levels of other large HSPs (HSP90, HSP70, and HSPA5) remained unchanged after IBMX induction (Fig. 1D). Because the temporal expression patterns of both HSP70 and HSP90 were different from that of HSP27 in this model, the role of HSP27 in the neuronal differentiation of PDMCs is likely to differ from those of other HSPs.

### Dynamic expression profile of HSP27 protein during mouse embryonic development

**Figure 2:**
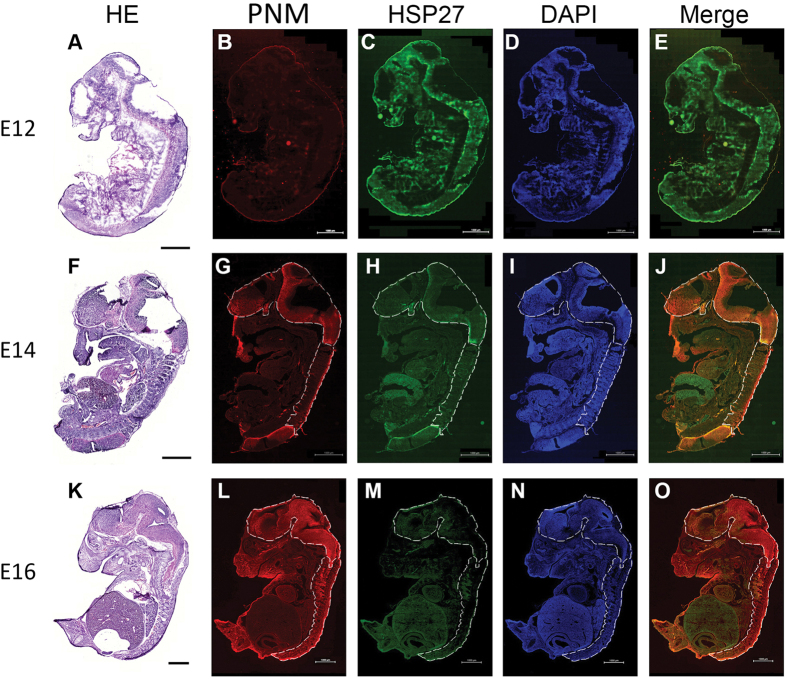
Localization of HSP27 protein and a Pan-Neuronal Marker reveals different spatial and temporal expression patterns during mouse embryo development. Cryosections from mouse embryos harvested at E12, E14 and E16 were stained with HE for embryo histology (**A,F,K**), Pan-Neuronal Marker (PNM) for identifying the distribution of neuronal cells (**B,G,L**), anti-HSP27 (**C,H,M**) and DAPI (**D,I,N**). Dashed white lines indicate the central nervous system. Initial staining (E12) with the PNM was very low (**B**) while HSP27 was distributed throughout the whole embryo. (**C**) PNM staining appeared at E14 (**G**), while HSP27 protein expression gradually centralized in particular organs in the embryo. (**H**) Colocalization was observed between HSP27 and PNM except in the gastrointestinal region. (**J**) At E16, PNM staining was extensive (**L**); however, the HSP27 expression levels in many tissues had decreased, especially in the central nervous system. (**M**) There were rare co-localizations between HSP27 and the PNM throughout whole section. The white bar represents 1000 μm.

To evaluate the protein expression of HSP27 during neuronal differentiation *in vivo*, we performed immunofluorescent staining of HSP27 at various stages of mouse embryonic development. The Pan-Neuronal Marker was used to visualize neurons. The fluorescent signal for HSP27 (green) decreased (Fig. 2C,H), whereas that of the Pan-Neuronal Marker (red) increased in the brain and spinal cord (Fig. 2B,G) from mouse embryonic day 12 (E12; Figure A to E) to E16 (Figure F to J). These findings demonstrated that the downregulation of HSP27 is correlated with neuronal differentiation *in vivo*.

### Overexpression of HSP27 suppresses neuronal differentiation in IBMX-treated PDMCs

**Figure 3:**
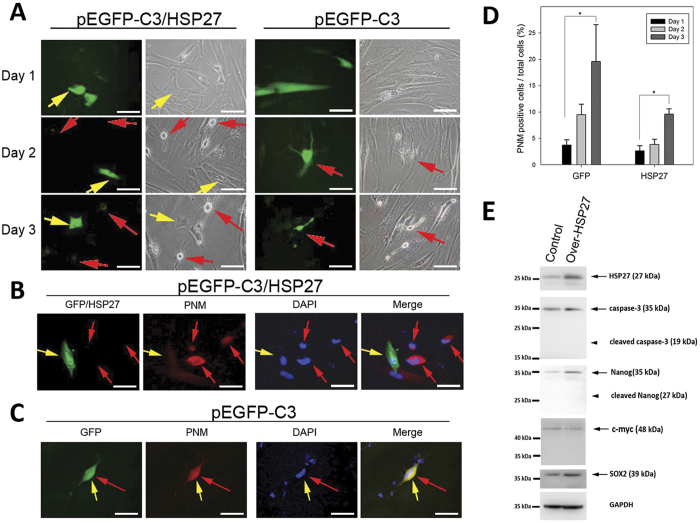
Physiological effects of over-expression of HSP27 in PDMCs. The PDMCs were induced with IBMX for 6 h and then transfected with the pEGFP-C3 or pEGFP-C3/HSP27 plasmids, as indicated. (**A**) The cells were fixed and directly visualized using fluorescence microscopy at 1 day, 2 days and 3 days after transfection. The green cells show the expression of GFP. The red arrows indicate the neuron-like cells, and yellow arrows indicate the GFP-expressing cells. (**B**) Cells from each transfection were fixed and probed with Pan-Neuronal Marker (PNM) antibody for visualizing neuronal cells. The GFP/HSP27-transfected cells showed green signal alone, without co-localization with PNM-positive cells. (**C**) Without HSP27 overexpression, there was partial colocalization of the GFP and PNM signals. (Magnification = 200x). (**D**) The transfected cells in each condition were probed with PNM antibody, and PNM fluorescence signals were recorded and quantified. The cells showing PNM immunofluorescence were counted as positive cells. The proportion of differentiated neurons was quantified as the positive PNM immunofluorescence staining divided by the DAPI-positive cells. (**E**) Immunoblots of PDMCs with HSP27 overexpression. The upregulated HSP27 protein expression levels were confirmed in HSP27-overexpressing cells (right column) compared with controls. HSP27-overexpressing PDMCs (indicated by arrowheads) showed no cleaved forms of caspase-3 or Nanog. Additionally, the cleaved forms of SOX2 and c-myc, two other stem cell markers, were absent in both experimental conditions. GAPDH was used as a loading control for immunoblotting.

To further characterize the role of HSP27 in the process of neuronal differentiation, we overexpressed green fluorescent protein (GFP) or a GFP/HSP27 fusion protein in PDMCs that were previously incubated with IBMX for 6 h. As shown in Fig. 3A (right panel), IBMX-induced PDMCs expressing GFP alone retained their ability to differentiate into neuron-like cells (red arrow), even in cases of overexpression of GFP and regardless of the duration of cell culture after transfection. Conversely, cells expressing the GFP/HSP27 fusion protein exhibited a non-neuronal morphology (Fig. 3A, left panel). We also probed transfected PDMCs with the Pan-Neuronal Marker antibodies (Fig. 3B,C). In Fig. 3B, the cell that exhibited GFP fluorescence also presented positive immunostaining for the Pan-Neuronal Marker (red). Concomitantly, the signals from the GFP/HSP27 fusion protein and from the Pan-Neuronal marker antibody (red) were detected in different cells (Fig. 3C). Notably, the cells positive for the Pan-Neuronal Marker exhibited a lower degree of HSP27 overexpression (Fig. 3D). In order to strengthen the results regarding the HSP27-silenced PDMCs (hereafter in the manuscript), immunoblotting for caspase-3 and stem cell markers such as Nanog, SOX2 and c-myc all appeared unchanged in the HSP27-overexpressed PDMCs, indicating that HSP27 expression is beneficial for sustaining the stemness of the PDMCs (Fig. 3E). These results suggest that the overexpression of HSP27 prevented the differentiation of IBMX-induced PDMCs into neurons.

### Knockdown of HSP27 expression enhances the ability of PDMCs to differentiate into neurons

**Figure 4:**
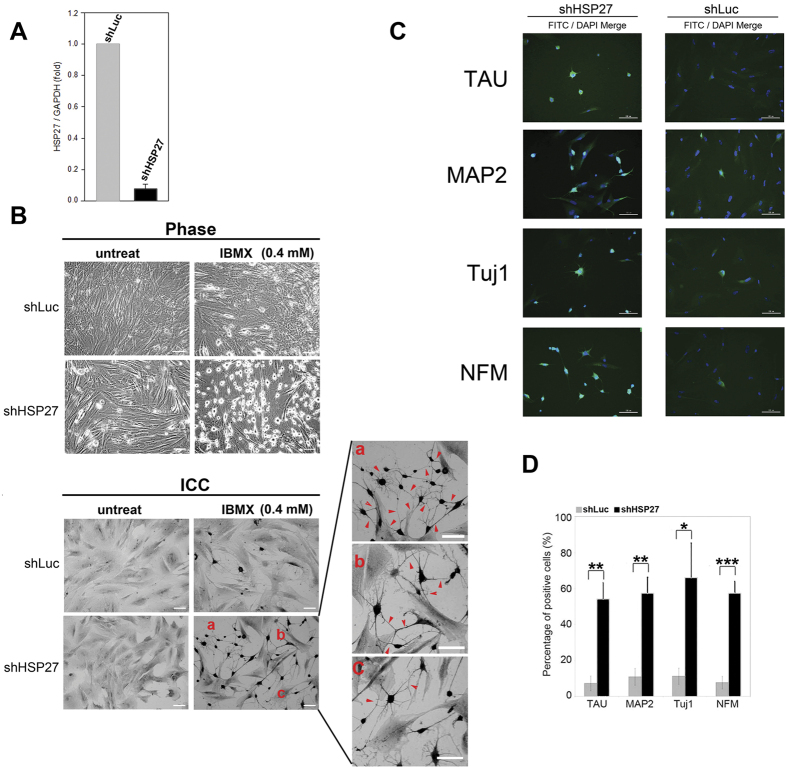
Characterization of neurons induced from HSP27-silenced PDMCs. PDMCs were infected with lentiviruses containing shRNAs targeting HSP27 (shHSP27) or luciferase (shLuc). After eighteen days of puromycin selection, the HSP27 mRNA expression levels were quantified by qRT-PCR (**A**). HSP27-silenced PDMCs exhibited 90% downregulation of HSP27 expression. The results were determined in three independent experiments. The HSP27-silenced and Luc-silenced PDMCs were treated with 0.4 mM IBMX. After 6 h, the cells were imaged using an inverted phase microscope (**B**, upper panel, Phase). The cells were also subjected to immunocytochemical staining for MAP2, a neuron-specific cytoskeletal protein (**B**, lower panel, ICC). The HSP27-silenced PDMCs and Luc-silenced PDMCs showed almost identical cell morphology before induction. However, after induction, the HSP27-silenced PDMCs showed numerous differentiated neurons compared to the Luc-silenced cells. Additionally, there were many synaptic connections, some of which had begun to form a network, among the HSP27-silenced PDMCs, as indicated by red arrowheads. The relative positions of the enlarged rectangles are indicated by a, b and c in the original image. (Scale bar = 100 μm). (**C**) The HSP27-silenced or Luc-silenced cells were induced with 0.4 mM IBMX and fixed for immunostaining. The cells were stained with primary antibodies against several neuronal markers including Tau, MAP2, Tuj1 and NFM, followed by appropriate FITC-conjugated secondary antibodies. The cell nuclei were counterstained with 4′,6-diamino-2-phenylindole (DAPI). The FITC and DAPI images for each condition were merged. (Scale bar = 100 μm). (**D**) The bar chart shows the percentage of FITC-positive cells among the DAPI-positive cells quantified from the above conditions. *p < 0.05; **p < 0.01; ***p < 0.001.

Next, we investigated the effects of the silencing of HSP27 in PDMCs. Eighteen days after shHSP27 infection, the knockdown of HSP27 by shRNA (shHSP27) yielded at least 90% inhibition of HSP27 mRNA expression (Fig. 4A). Compared with the control (shLuc) infection, neuron-like cells were dominant in the shHSP27-infected PDMCs under induction (Fig. 4B, Phase, right lower panel). Differences in immunostaining were also detected using an anti-NSE antibody. As depicted in Fig. 4B, numerous neuron-like cells were detected, and many of them were connected with synapse and formed networks (Fig. 4B, ICC, right panel, red arrowheads). To confirm the neuronal characteristics of the cells, four additional neuron-specific antibodies (TAU, MAP2, Tujl, and NFM) were used to probe these neuron-like cells (Fig. 4C). Compared with the shLuc-infected PDMCs (Fig. 4C, right panel), the neuron-specific markers examined showed significantly elevated expression in HSP27-silenced PDMCs, indicating the presence of neuronal differentiation (Fig. 4C, left panel). For each antibody, the immunopositive cells were quantified and normalized, and staining for each neuronal marker was significantly stronger in the HSP27-silenced cells (Fig. 4D). HSP27-silenced and Luc-silenced PDMCs after induction were also observed via time-lapse imaging (Video 1). Sequential changes in neuronal morphology were observed from the videos; in HSP27-silenced PDMCs, the onset of neuronal differentiation was earlier than that detected in Luc-silenced cells after induction, and the number of neuron-like cells was greater in HSP27-silenced than in Luc-silenced PDMCs.

### Cellular effects after HSP27 knockdown in PDMCs

**Figure 5:**
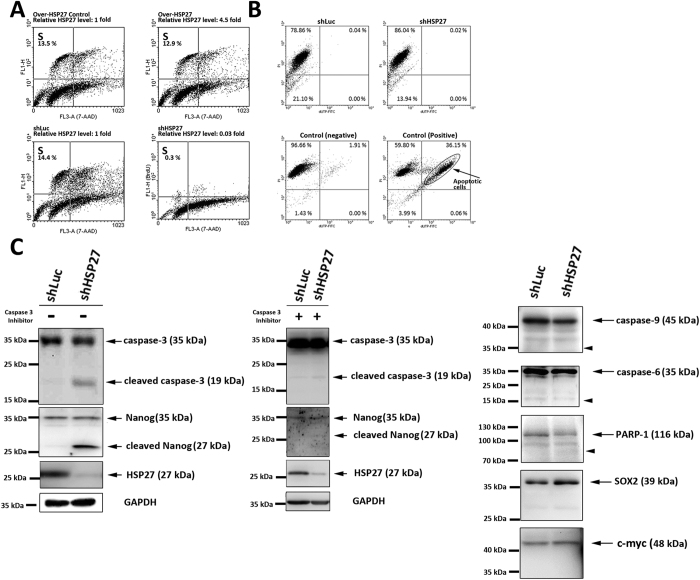
Cellular effects of HSP27-silenced PDMCs are associated with cleavage of caspase-3 and Nanog. PDMCs were infected with shRNA-containing lentiviruses targeting luciferase or HSP27 (shLuc or shHSP27). After eighteen days of selection with puromycin, the cells were harvested for the following experiments. (**A**) Changes in HSP27 expression levels in the PDMCs do not induce apoptosis. Apoptosis was examined using the Apo-Direct TUNEL assay. Neither shLuc nor shHSP27 infection of PDMCs produced TUNEL-positive cells compared with the apoptotic positive control cells. (**B**) Downregulation of HSP27 expression arrests the cell cycle before entering S phase. To test the cellular effects after manipulation of HSP27, we performed a BrdU incorporation assay and calculated the percentages of cells in S phase within HSP27-overexpressing and HSP27-silenced PDMCs. HSP27-overexpressing PDMCs, which contained 4.5 times the control level of HSP27, showed BrdU incorporation with relative the same rate observed in the pLKO transient transfection control. However, the BrdU incorporation decreased to 0.3% of the control level in the HSP27-silenced PDMCs cells, which had a relative HSP27 expression level of 0.03 compared to the shLuc infection control group. The inhibition of BrdU incorporation in the HSP27-silenced PDMCs cells suggested cell cycle arrest at the G1 to S phase transition associated with HSP27 downregulation. (**C**) Immunoblots of shLuc- or shHSP27-infected PDMCs with or without Z-DEVD-FMK treatment. Among the PDMCs incubated without Z-DEVD-FMK, a caspase-3 inhibitor, the cleaved forms of caspase-3 and Nanog were detected in HSP27-silenced PDMCs (left column). For the caspase-3-inhibitor treatment, 50 μM Z-DEVD-FMK was applied to shLuc- or shHSP27-infected PDMCs and re-applied every two days until harvest. Faint bands for cleaved caspase-3 and Nanog were observed in HSP27-silenced PDMCs (middle column). The downregulation of HSP27 protein expression was confirmed in HSP27-silenced cells. In addition, SOX2 and c-myc, two other stem cell markers, were not cleaved in either experimental condition. Other apoptosis-related enzymes, caspase-9, caspase-6, caspase-8 and PARP-1, showed no existence of executioner forms (right column, indicated by arrowheads). GAPDH was used as a loading control for immunoblotting.

To clarify the cellular and molecular events induced by HSP27 depletion, the HSP27 expression levels were manipulated in PDMCs without IBMX induction in this section and the next. Compared with the pLKO transient transfection control (Fig. 5A, left upper panel), we found that BrdU incorporation varied little in the PDMCs overexpressing HSP27 (overHSP27), which showed HSP27 expression at 4.5 times that in the control (Fig. 5A, right upper panel). The elevation of the level of expression of HSP27 did not alter the percentage of cells in the S phase. However, BrdU incorporation decreased from 14.4% to 0.3% in the HSP27-silenced (shHSP27) PDMCs, which showed HSP27 expression levels at 3% of that in the shLuc controls (Fig. 5A, right lower panel and left lower panel, respectively). In short, the ability to incorporate BrdU was greatly inhibited in shHSP27-infected PDMCs, indicating that the cell cycle arrested before entering the S phase in HSP27-silenced stem cells.

### Molecular effects of HSP27 knockdown in PDMCs

In addition to the reduction of BrdU incorporation, the rate of apoptosis was not increased in HSP27-silenced PDMCs (Fig. 5B, right upper panel) compared with shLuc-infected PDMCs (Fig. 5B, left upper panel) and the apoptosis-negative control cells (Fig. 5B, left lower panel). This suggests that the physiological role of HSP27 protein when PDMCs exit the cell cycle before the S phase is not anti-apoptotic. Moreover, the presence of active (cleaved) caspase-3 was found in HSP27-silenced PDMCs (Fig. 5C, left column). However, the activated caspase-3 did not induce apoptosis in these cells (Fig. 5B, right upper panel). Subsequently, Nanog expression was evaluated in HSP27-silenced PDMCs (Fig. 5C, left column). Nanog was cleaved when HSP27 protein expression was low and a proportion of caspase-3 was activated. To identify the specific pathway derived from HSP27 downregulation, 50 μM Z-DEVD FMK, a caspase-3 inhibitor, was applied to shLuc- or shHSP27-infected PDMCs. The immunoblots showed faint bands for cleaved caspase-3 and cleaved Nanog in shHSP27-infected PDMCs with caspase-3 inhibitor incubation, demonstrating that the Nanog cleavage observed in HSP27-silenced cells was mediated by caspase-3 activation (Fig. 5C, middle column). Other stem cell markers, including SOX2 and c-myc (Fig. 5C, right column), were not cleaved. Additionally, the active forms of other apoptosis-related proteins, including caspase-9, caspase-6, and PARP-1, were not observed in HSP27-silenced cells (Fig. 5C, right column, indicated by the arrowhead). Briefly, the cleaved forms of caspase-9, caspase-6, PARP-1, SOX2 and c-myc were not detected in HSP27-silenced PDMCs, even in cells that contained cleaved caspase-3, indicating that the specific pathway by which HSP27 downregulation induced Nanog cleavage was mediated by caspase-3.

### Induced neurons derived from HSP27-silenced PDMCs can respond to classical activators of glutamatergic neurons

**Figure 6:**
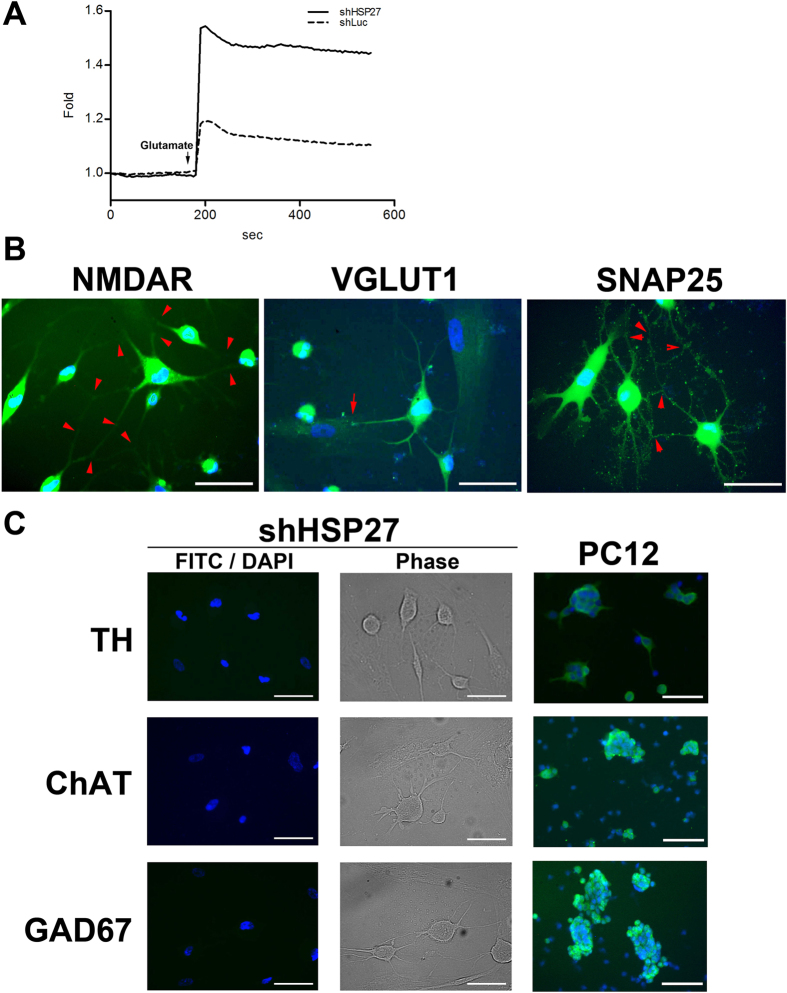
Knockdown of HSP27 directs PDMCs to differentiate into glutamatergic neurons. (**A**) Neuronal function in induced, HSP27-silenced PDMCs was determined based on Ca^2+^ influx profiles. The arrow indicates the addition of glutamate (20 μM). The plot shows the higher-magnitude kinetic profile in HSP27-silenced PDMCs after glutamate addition compared with Luc-silenced cells. (**B**) Immunofluorescent analysis of shHSP27-infected PDMCs induced with 0.4 mM IBMX. The cells were stained with primary antibodies against the glutamatergic neuron markers NMDAR, VGLUT1 and SNAP25, followed by the appropriate FITC-conjugated secondary antibodies. The cell nuclei were counterstained with 4′,6-diamino-2-phenylindole (DAPI). The attachment of a dendrite from another cell (red arrow) and synaptic connections (red arrowhead) are indicated. (**C**) Induced neurons derived from HSP27-silenced cells were stained for a dopaminergic neuron marker, TH; a cholinergic neuron marker, ChAT; and a GABAergic neuron marker, GAD67. The FITC and DAPI images for each condition were merged. The phase contrast images of each condition are also shown to demonstrate the neuronal phenotype of each staining. PC12 cells were used as positive controls for each antibody. (Scale bar = 100 μm). Abbreviations: N-methyl-D-aspartate receptor, NMDAR; vesicular glutamate transporter 1, VGLUT1; synaptosomal-associated protein 25, SNAP25; tyrosine hydroxylase, TH; choline acetyltransferase, ChAT; and glutamic acid decarboxylase, GAD67.

To investigate the function of induced neurons derived from IBMX-induced HSP27-silenced cells, we evaluated the calcium influx by imaging calcium signalling in response to glutamate, the primary agonist of the NMDA receptor. Differentiated HSP27-silenced cells were treated with 20 μM glutamate. The neuronal function was measured as the change in [Ca^2+^]_*i*_ after glutamate stimulation. The stimulation produced large and immediate calcium increases (Fig. 6A), which indicated membrane depolarization and thus calcium flux through calcium channels in the induced neurons after glutamate stimulation. The [Ca^2+^]_*i*_ increased more strongly in the HSP27-silenced PDMCs than in the Luc-silenced PDMCs because there were many more neurons in the population of HSP27-silenced PDMCs. We also performed time-lapse imaging after glutamate stimulation. Several neuron cells showed intermittent Ca^2+^ influx and efflux (Video 2, red arrow), demonstrated that there were not only functional Ca^2+^ influx channels in the presynaptic nerve terminals, serving as the gate for controlling Ca^2+^ influx, but also plasma membrane Ca^2+^ pumps, which generated Ca^2+^ efflux from the induced neuron. These findings implied the transmission of signals from one functional glutamatergic neuron to other neurons through synaptic connections, causing the Ca^2+^ influx, followed by the absence of such a signal, resulting in Ca^2+^ efflux.

### HSP27-silenced PDMCs differentiated into the glutamatergic neuron lineage

We next used immunofluorescent analysis to investigate the presence of markers of specific neuron lineages, including N-methyl-D-aspartate receptor (NMDAR, glutamatergic), vesicular glutamate transporter 1 (VGLUT1, glutamatergic) and synaptosomal-associated protein 25 (SNAP25, glutamatergic), tyrosine hydroxylase (TH, dopaminergic), choline acetyltransferase (ChAT, cholinergic) and glutamic acid decarboxylase (GAD67, GABAergic). Among them, only markers of glutamatergic neurons showed positive staining (Fig. 6B). NMDA receptors are activated by the binding of glutamate and glycine and allow calcium influx through the cell membrane. VGLUT1 protein is involved in the formation of synaptic vesicles, specifically in glutamate transport. SNAP25 is a t-SNARE protein that specifically participates in membrane fusion of synaptic vesicles with the plasma membrane. Notably, the NMDAR-, VGLUT1- or SNAP25-positive neurons exhibited many synaptic connections with each other (Fig. 6B, red arrow). However, markers of other lineages were absent in the HSP27-silenced neurons (Fig. 6C, FITC/DAPI panel), although there were neuron differentiations (Fig. 6C, Phase panel). Combining the immunofluorescence and [Ca^2+^]_*i*_ flux data suggests that silencing of HSP27 enhanced the differentiation of PDMCs into functional glutamatergic neurons.

## Discussion

The benefits of stem cells in clinical applications include their self-renewal properties and their capacity to differentiate into various types of cells. Therefore, the neural differentiation of PDMCs may signal a new era in the field of neuronal regeneration, for clinical applications beyond the limitations of tissue sources^7^. Complex regulation of neural differentiation and neurite outgrowth, in a spatially and developmentally regulated manner, is essential for the formation of specific neurons^22^, and based on our results, the induced neurons derived from HSP27-silenced PDMCs showed numerous synaptic connections and even formed networks, indicating differentiation into mature neurons.

HSP27 is known to have multiple functions. In addition to chaperone activity, this protein regulates cytoskeletal dynamics^23^ and is involved in cell-cycle regulation^14^. Mutated HSP27 leads to an irregular distribution of the neurofilament light-chain protein^24^. Furthermore, HSP27 may block caspase activation to inhibit apoptosis^25^. Although the association between decreased HSP27 expression and neurite outgrowth has been reported elsewhere^26^, we were the first to explore the downregulation of HSP27, at both the protein and mRNA levels, *in vivo* and *in vitro*. In the present study, the expression of HSP27 was markedly reduced during neural differentiation. *In vivo*, the reversal of the expression of HSP27 and the neural markers observed during mouse embryonic development suggests that the downregulation of HSP27 is critical for neural differentiation. The crucial role of HSP27 in neural differentiation was also detected after the manipulation of HSP27 expression *in vitro*. First, the positive immunostaining for various neural markers was observed when the neurites grew after HSP27 knockdown. Second, the overexpression of HSP27 clearly suppressed neural differentiation. Taken together, these results suggest that HSP27 plays a key role in the process of neural differentiation.

As reported by Launay *et al*., cellular differentiation shares some physiological processes with apoptosis, in a decision between cell death or differentiation^27^. Some specific differentiation procedures must occur simultaneously with precise apoptosis for cell-fate commitment^28^. Therefore, the precise regulation of the activities of caspases is critical in differentiation models^29^, and HSPs might be essential to the orchestration of the decision between apoptosis and differentiation^14^. In fact, the function of activated caspase-3 is not restricted to the execution of apoptosis; it also participates in cell proliferation^30^, erythroid maturation^31^, skeletal muscle differentiation^32^, osteoblastic differentiation^33^, and stem cell differentiation^34^,^35^. The activation of caspase-3 was negatively regulated by HSP27^36^,^37^. This is consistent with our results that the cleavage of caspase-3 and Nanog can facilitate neural differentiation from PDMCs because of the downregulated HSP27. Moreover, the cells with HSP27 knockdown showed no cleaved forms of SOX2 and c-myc, demonstrating that the specific pathway begins at HSP27 and continues to Nanog and then caspase-3. Therefore, we propose that HSP27 must act upstream of those sequential cellular responses during neural differentiation.

The results obtained for invariable HSPs (HSP70, HSP90, and HSPA5), initiator caspases (caspase-9 and caspase-6), and the uncleaved form of PARP-1 suggest that apoptosis does not occur in HSP27-silenced PDMCs, even though caspase-3 is activated during the process of neural differentiation. Therefore, whether and how the actual apoptotic pathway is shut down when development is complete requires further investigation.

In glutamatergic neurons, synaptic vesicles are loaded with the excitatory neurotransmitter glutamate by VGLUT1, which generates glutamate uptake into intracellular vesicles^38^. SNAP25 participates in forming the SNARE complex, which is essential for the exocytosis of synaptic vesicles^39^, and it has been reported that GABAergic synapses, both in culture and in brain, lack SNAP-25 expression^40^. Based on our results, the positive immunostaining for NMDAR, VGLUT1 and SNAP25, as well as the elevated calcium influx after NMDA or glutamate stimulation, demonstrate that the induced neurons show characteristics of glutamatergic neurons. In addition, the positive staining for SNAP25 and negative staining for GAD67 excluded the possibility that the HSP27-silenced PDMCs had differentiated into GABAergic neurons.

There are several mechanisms that control intracellular Ca^2+^ concentration, including voltage-gated channels^41^, ligand-gated channels^42^, and IP_3_R-controlled release from the endoplasmic reticulum^43^. According to our results, HSP27-silenced PDMCs showed calcium influx and efflux after stimulation with excitatory molecules, indicating the expression of voltage-dependent channels and [Ca^2+^] transporters in the induced neurons. In hippocampal pyramidal neurons, glutamate is the major transmitter, and the resulting calcium flux through specific channels is thought to be critical for cognitive functions such as learning and memory via long-term potentiation and synaptic plasticity^44^. Loss of pyramidal neurons and their synapses, resulting in glutamatergic hypoactivity, has been reported in Alzheimer’s disease^45^. Moreover, ionotropic glutamate receptor reprogramming has been reported as an important factor in some types of retinal degeneration^46^, and in a parallel submission, we found that suppression of HSP27 expression contributes to restoring retinal function after light-induced retinal degeneration. It is worth investigating the mechanism underlying the functional restoration of retinal and glutamatergic neuron differentiation in retinal degeneration-related diseases.

**Figure 7:**
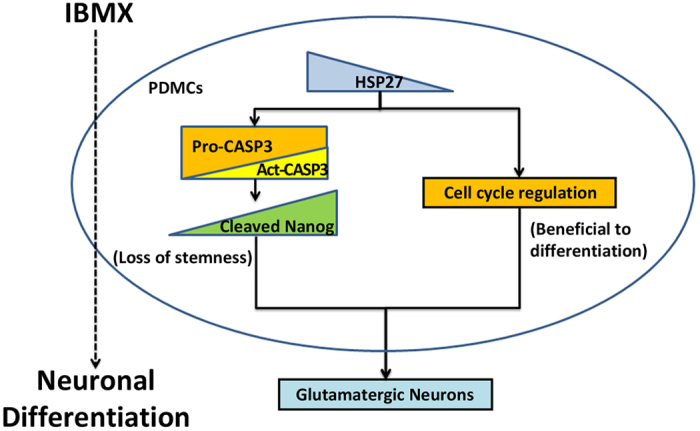
A proposed model of HSP27-blockaded glutamatergic neuron differentiation from PDMCs. In undifferentiated PDMCs, HSP27 protein is expressed at normal levels. After the addition of IBMX, the protein level of HSP27 decreased; pro-caspase 3 then auto-cleaved into activated caspase-3 (Act-CASP3), which then cleaves Nanog, causing PDMCs to lose their stemness. The downregulation of HSP27 prevents the cell cycle from entering S phase, which is also beneficial for differentiation. Both effects derived from the downregulation of HSP27 promote the differentiation of PDMCs into glutamatergic neurons.

In conclusion, our study demonstrates a novel regulatory mechanism of HSP27 in the differentiation of glutamatergic neurons derived from PDMCs. A proposed model is shown in Fig. 7. In undifferentiated PDMCs, HSP27 protein associates with pro-caspase-3 (Pro-CASP3) and maintains the progression of the cell cycle to continue stem cell proliferation. After the addition of IBMX, the HSP27 protein level decreases, and HSP27 dissociates from pro-caspase-3, resulting in free pro-caspase-3, which undergoes auto-cleavage into activated caspase-3 (Act-CASP3). The activated caspase-3 then cleaves Nanog, resulting in PDMCs losing their stemness. The downregulation of HSP27 prevents the cell cycle from entering S phase, which is also beneficial for differentiation. Both effects derived from the downregulation of HSP27 promote the differentiation of PDMCs into glutamatergic neurons. The precise control of HSP27 expression must be spatially and temporally regulated together with anti-apoptosis signals during neural differentiation. Our findings suggest that an agent or procedure that can manipulate HSP27 expression will be a potential target for future drug development in the field of neurodegenerative disease.

## Materials and Methods

### PDMC culture and neuronal differentiation

PDMCs were obtained as described previously^47^, with some modifications. The healthy donors provided informed consent, and the study protocol was approved by the Institutional Review Board of the Cathay General Hospital. The protocol was carried out in accordance with the approved guidelines. Term placentas were cut using sterilized scissors, and the harvested pieces of tissue were washed with phosphate-buffered saline and digested with 0.25% trypsin–EDTA (Gibco/Life Technologies, Carlsbad, CA, USA) for 10 min at 37 °C. Subsequently, the homogenate was centrifuged and resuspended in complete Dulbecco’s Modified Eagle’s Medium (DMEM, Gibco/Life Technologies, Carlsbad, CA, USA) with 10% fetal bovine serum (FBS, HyClone/GE Healthcare, Novato, CA, USA), 100 U/mL penicillin, and 100 g/mL streptomycin (Sigma-Aldrich, St. Louis, MO, USA). Cell cultures were maintained at 37 °C with 5% CO_2_. For the neuronal differentiation of PDMCs, cells were incubated with 0.4 mM IBMX in complete DMEM for 3 days, as reported previously^10^. For Caspase-3 inhibition treatment, 50 μM of Z-DEVD-FMK (#SC-311558A, Santa Cruz Biotechnology, Dallas, TX, USA) was incubated with the cells and re-added after two days during the experiments.

### Immunocytochemistry

Cultured cells were fixed with 4% paraformaldehyde for 5 min at room temperature and permeabilized with 0.1% Triton-X 100 for 20 min. A primary antibody against NSE (1:25, #SAB4200571, Sigma-Aldrich, St. Louis, MO, USA) was applied, followed by incubation with a biotinylated anti-rabbit antibody and a conjugate of avidin–biotin with horseradish peroxidase (#A-2004, Vector Laboratories, Burlingame, CA, USA). The slides were treated with the Vector VIP substrate kit (#SK-4600, Vector Laboratories, Burlingame, CA, USA), to visualize the resulting peroxidase activity. The cellular nucleus was counterstained with hematoxylin.

### Two-dimensional gel electrophoresis (2-DE)

Lysates of PDMCs treated with IBMX for 0, 24, and 48 h were prepared for 2-DE using a method reported previously, with slight modifications^10^. The details of 2-DE procedures and protein identification methods were described in Supplement file.

### Total RNA extraction and quantitative real-time PCR

The total RNA from PDMCs treated with or without IBMX was independently extracted using the RNeasy Mini Kit according to the manufacturer’s instructions (Qiagen, Hilden, Germany). Before qRT–PCR, each extract of total RNA was quantified using a NanoDrop ND-1000 spectrophotometer (Thermo Fisher Scientific, Waltham, MA, USA) and qualified using a Bioanalyzer RNA Nano 6000 chip (Agilent Technologies, Santa Clara, CA, USA) according to the manufacturer’s instructions. The relative levels of the HSP27 mRNA were quantified using qRT–PCR in the presence of a TaqMan probe and the TaqMan Master Mix (Roche Diagnostics GmbH, Mannheim, Germany), according to the manufacturer’s instructions. The primers used for the amplification of HSP27 (NM_001540) were 5′–CCCTGGATGTCAACCACTTC–3′ (forward) and 5′–GATGTAGCCATGCTCGTCCT–3′ (reverse), together with Universal Probe No. 22 (Roche Diagnostics GmbH, Mannheim, Germany). For each quantification assay, the level of expression of the HSP27 mRNA was normalized by dividing the result by the level of expression of the 18s rRNA. The LightCycler Software (Version 4.05, Roche Diagnostics GmbH, Mannheim, Germany) was used to generate quantitative data^48^. To calculate the relative expression of HSP27 in each IBMX-treated PDMC population, we calculated the ratio of HSP27 mRNA expression in PDMCs at different times after IBMX induction.

### Immunoblotting

Immunoblot analysis was performed according to previous protocols, with minor modifications^49^. Aliquots of equivalent cell-lysate proteins were subjected to 12% SDS–PAGE and transblotted onto a polyvinylidine fluoride membrane (25 mM Tris [pH 8.3], 192 mM glycine, 0.1% SDS, and 15% methanol) using a Semidry Transphor unit (Nihon Eido, Tokyo, Japan) at a constant current of 60 mA for 1 h. After the transfer, the membrane was blocked with 5% defatted milk in TBST buffer (20 mM Tris [pH 7.4], 150 mM NaCl, and 0.05% Tween 20) for 1 h. The membrane was first probed with primary antibodies in TBST buffer for 1 h. The primary antibodies and dilutions used were as follows: anti-human HSP27 (1:1,000, #ab1426; Abcam, Cambridge, UK), anti-HSP90 (1:1,000, #ab54285; Abcam, Cambridge, UK), anti-HSP70 (1:1000, #ab47455; Abcam, Cambridge, UK), anti-HSPA5/GRP78 (1:1000, # ab137404; Abcam, Cambridge, UK), anti-caspase 3 (1:1,000, #9662S; Cell Signaling, Danvers, MA, USA), anti-Nanog (1:2,000, #AB9220; Chemicon/Merck Millipore, Billerica, MA, USA), anti-SOX2 (1:1,000, #20118-1-AP; Proteintech, Chicago, IL, USA), anti-c-myc (1:1000, # ab51154; Abcam, Cambridge, UK), anti-caspase 9 (1:1,000, #9502; Cell Signaling, Danvers, MA, USA), anti-PARP-1 (1:1,000, #9542; Cell Signaling, Danvers, MA, USA), anti-caspase 6 (1:1,000, #9762; Cell Signaling, Danvers, MA, USA), anti-caspase 8 (1:500, #9496; Cell Signaling, Danvers, MA, USA), and anti-GAPDH (1:2,000, #AM4300; Ambion/Life Technologies, Austin, TX, USA). The membrane was then incubated with horseradish-peroxidase-conjugated anti-rabbit or anti-mouse antibodies (1:5,000, #A0545 or #A4416; Sigma-Aldrich, St. Louis, MO, USA). Protein signals were detected using the Western Lightning Chemiluminescence Reagent Plus (PerkinElmer, Waltham, MA, USA).

### Detection of HSP27 expression at different stages of mouse embryonic development

Mice were maintained in a controlled environment, and E12–E16 embryos were obtained from Balb/c females that were mated with Balb/c males. The stages of embryonic development were determined based on morphology. After harvest, embryos were fixed with 4% paraformaldehyde in phosphate-buffered saline (PBS) for 10 min and then washed with PBS three times. The detailed procedures of embryo cryosection, immunostaining and visualization of HSP27 are described in Supplement file.

### Transient expression and silencing of HSP27 in PDMCs

To overexpress HSP27 in PDMCs, the *HSP27* gene (human placental cDNA; Sigma-Aldrich, St. Louis, MO, USA) was cloned into pEGFP-C3 (Clontech Laboratories, Mountain View, CA, USA). The integrity of sequences was confirmed by DNA sequencing. PDMCs in the logarithmic growth phase were treated with IBMX for 6 h. Subsequently, a fusion construct of green fluorescent protein (GFP) and HSP27, or GFP alone, was transiently expressed in PDMCs for 2 days using the Fugene HD reagent (Roche Diagnostics GmbH, Mannheim, Germany). To knock down HSP27 in PDMCs, pLKO.1-puro plasmid-based short-hairpin RNAs (shRNAs), including shLuc (luciferase shRNA) and shHSP27 (HSP27 shRNA), were obtained from the National RNAi Core Facility (Academia Sinica, Taipei, Taiwan). Purified shLuc and shHSP27 shRNA plasmids were then separately transfected into H293T cells using Lipofectamine (Invitrogen/Life Technologies, Carlsbad, CA, USA) together with an envelope expression plasmid (pMD2.G) and a packaging vector (psPAX2), to generate shRNA-containing lentiviruses. Each lentivirus was then infected into PDMCs according to the National RNAi Core Facility protocol. Stable clones expressing shRNAs were selected using puromycin (0.5 μg/mL; Calbiochem/Merck Millipore, San Diego, CA, USA). The relative levels of the HSP27 mRNA were quantified using qRT–PCR, as described previously.

### Immunofluorescence and quantification of neuronal differentiation

Cultured cells were fixed and blocked as described previously^11^. Subsequently, the cells were incubated with primary antibodies individually overnight at 4 °C. The brand and dilution of the primary antibodies were as follows: Pan-Neuronal Marker (1:100, MAB2300; Merck Millipore, Billerica, MA, USA), anti-TAU (1:100, ab80579; Abcam, Cambridge, UK), anti-MAP2 (1:200, AB5622; Chemicon/Merck Millipore, Billerica, MA, USA), anti-Tujl (1:50, MAB1637; Chemicon/Merck Millipore, Billerica, MA, USA), anti-NFM (1:40, N5264; Sigma-Aldrich, St. Louis, MO, USA), anti-GAD67 (1:100, AB26116, Abcam, Cambridge, UK), anti-NMDAR2B (1:200, AB65783, Abcam, Cambridge, UK), anti-VGLUT1 (1:200, AB72311, Abcam, Cambridge, UK), anti-SNAP25 (1:100, AB109105, Abcam, Cambridge, UK), anti- ChAT (1:1000, AB68779, Abcam, Cambridge, UK), anti-TH (1:500, ARG52461, ARGIO, Hsinchu City, Taiwan). The cells were then incubated with appropriate fluorescein-labeled secondary antibodies for 1 h at room temperature. Before visualization, 4′,6′-diamidino-2-phenylindole (DAPI) was added to stain cell nuclei. All images were acquired using a Nikon Eclipse 80i fluorescence microscope (Nikon Instruments, Tokyo, Japan). To evaluate neuronal differentiation, the percentage of differentiated neurons was calculated as the number of cells with TAU-, Neuro D-, MAP2-, Tuj1-, or NFM-positive signals over the number of DAPI-positive cells. The live-cell images were captured by the time-lapse photography of laser scanning confocal microscopy Zeiss LSM 5 PASCAL (Carl Zeiss GmbH, Jena, Germany).

### Ca^2+^ influx measurement

Ca^2+^ influx measurement was carried out by spectrophotometer and laser scanning confocal microscopy. Cells were first induced with IBMX (0.4 mM) and then loaded with the calcium indicated dye Fluo-4 AM (5 μM, #F14217, Invitrogen) for 1 hr at 37 °C in a dark incubator. After that the medium were replaced by depolarization solution, which contained potassium chloride (90 mM) and ionomycin (2 μM, #I0634, Sigma-Aldrich) in DMEM. Cells were then place in the laser scanning confocal microscopy (Zeiss LSM 5 PASCAL, Carl Zeiss GmbH, Jena, Germany) for live-cell images or Synergy HT Multi-Mode microplate reader (BioTek, Winooski, VT, USA) for calcium influx measurement. Glutamate (#G1251, Sigma-Aldrich) was then added and the cells were stimulated with 488 nm wavelength and the emission light was filtered by 530 nm filter. Data was acquired every 5 second.

### BrdU incorporation assay

To test the cellular effects of HSP27 manipulation, PI-stained DNA and BrdU incorporation analyses were performed. BrdU (10 μM) was pulse incorporated into HSP27-overexpressing or HSP27-silenced PDMCs for 24 h using the APO-DIRECTkit (BD Biosciences, San Jose, CA, USA), according to the manufacturers’ protocol. The fixed cells underwent a 30 min incubation in PBS containing 0.2 mg/mL RNase A, 0.1% Triton X-100, and 20 μg/mL PI at room temperature. The fluorescent light emitted at 585 nm from PI-stained DNA and at 530 nm from BrdU–FITC-positive DNA was detected using a FACScan flow cytometer (BD Biosciences). The percentages of cells detected were determined using the FlowJo 8.7 software.

### Statistical analyses

Statistical analyses were performed using SPSS 13.0 for Windows (IBM SPSS, Armonk, NY, USA). Student’s *t*-test was used to compare the differences in the relative expression of HSP27 between the various IBMX-treated PDMC populations. Significance was set at *P* < 0.05. The data shown are representative of at least five experiments, with comparable results.

## Additional Information

**How to cite this article**: Cheng, Y.-C. *et al*. Knocking down of heat-shock protein 27 directs differentiation of functional glutamatergic neurons from placenta-derived multipotent cells. *Sci. Rep.* **6**, 30314; doi: 10.1038/srep30314 (2016).

## Supplementary Material

Supplementary Video 1

Supplementary Video 2

Supplementary Information

## Figures and Tables

**Table 1:** Identified protein of differentially expressed during IBMX induced neuronal differentiation.

Protein name	Coverage		Residue^b^	m/z
	aCoverage (%)	Peptide		
Heat shock 27	24.40%	(R)VPFSLLR(G)	6–12	831.5
		(R)LFDQAFGLPR(L)	28–37	1163.6
		(R)VSLDVNHFAPDELTVK(T)	97–112	1783.9
		(K)LATQSNEITIPVTFESR(A)	172–188	1906

^a^Percentage of total amino acid count to the mature protein.

^b^Amino acid sequence numbering according to the mature protein.
