# Corrigendum: Knocking down of heat-shock protein 27 directs differentiation of functional glutamatergic neurons from placenta-derived multipotent cells

**DOI:** 10.1038/srep32907

**Published:** 2016-09-14

**Authors:** Yu-Che Cheng, Chi-Jung Huang, Yih-Jing Lee, Lu-Tai Tien, Wei-Chi Ku, Raymond Chien, Fa-Kung Lee, Chih-Cheng Chien

Scientific Reports 6: Article number: 3031410.1038/srep30314; published online: 07 22 2016; updated: 09 14 2016

In the original version of this Article, Fa-Kung Lee and Chih-Cheng Chien were incorrectly affiliated with ‘Center for Biocellular Engineering, National Central University, Jhongli, Taiwan’. The correct affiliations are listed below.

Fa-Kung Lee

School of Medicine, Fu Jen Catholic University, New Taipei City, Taiwan.

Department of Obstetrics and Gynecology, Cathay General Hospital, Taipei, Taiwan.

Chih-Cheng Chien

School of Medicine, Fu Jen Catholic University, New Taipei City, Taiwan.

Department of Anesthesiology, Cathay General Hospital, Taipei, Taiwan.

These errors have now been corrected in the PDF and HTML versions of the Article.

