# Supplementary material for: Knocking down of heat-shock protein 27 directs differentiation of functional glutamatergic neurons from placenta-derived multipotent cells
Source: Sci Rep. 2016 Jul 22;6:30314. doi: 10.1038/srep30314 (PMC4957209; doi:10.1038/srep30314)
Supplement: Supplementary Information [file srep30314-s3.doc]

# Title: Knocking down of Heat-shock Protein 27 directs differentiation of functional glutamatergic neurons from Placenta-derived Multipotent Cells

***Short title: HSP27 level is critical for glutamatergic neuron differentiation***

**Author list: Yu-Che Cheng, Chi-Jung Huang, Yih-Jing Lee, Lu-Tai Tien,** **Wei-Chi Ku, Raymond Chien, Fa-Kung Lee and Chih-Cheng Chien**

**Supplement**

**Materials and Methods**

**Procedures of 2DE-PAGE**

Samples containing equivalent amounts of total proteins were mixed with 2-DE sample buffer (6 M urea, 2 M thiourea, 2% [w/v] CHAPS, 2% pharmalyte [pH 3–10], 60 mM DTT, and a trace of bromophenol blue) and were then rehydrated using Immobiline DryStrip gels (18 cm in length) with a pH gradient of 3–10 NL. The first dimension of 2-DE was performed on an ETTAN IPGphor II system (GE Healthcare Bio-Sciences, Piscataway, NJ, USA). The voltage gradients were as follows: 500 V for 0.5 kVh, gradient to 1,000 V for 1 kVh, gradient to 8,000 V for 24 kVh, and 8,000 V for 21.3 kVh, at a constant temperature of 20 °C. After IEF, the strips were equilibrated in an equilibration solution (50 mM Tris-HCl [pH 8.8], 6 M urea, 30% glycerol, 2% sodium dodecyl sulfate [SDS], and 0.01% bromophenol blue) with 100 mM DTT for 15 min at 25 °C. The strip was then reacted with 100 mM iodoacetamide for another 15 min and placed on top of a 12% SDS–polyacrylamide gel (size, 24 × 24 cm). The electrophoresis was performed at a constant voltage of 60 V using a vertical electrophoresis unit (Nihon Eido, Tokyo, Japan). The gels were stained using the mass compatible silver-staining method [14]. The images were captured using an image scanner with a 300 dpi resolution. The digitized images were then analyzed using PDQuest, Version 8.0.1 (Bio-Rad, Hercules, CA, USA).

## Protein identification

The protein spots of interest were excised and digested as described previously . The MS operation was performed as described previously. The digested peptides were resuspended in 20% formic acid. Peptide mass was determined using the positive ion mode of an LTQ-ion trap-Nano-LC-MS/MS apparatus (Thermo Fisher Scientific, Waltham, MA, USA). The identity of the proteins was determined after comparison of the LC-MS/MS data with the Swiss-Port database using the XCalibar software provided by Thermo Fisher Scientific.

**Embryo cryosection, immunostaining and visualization of HSP27**

The embryos were first submerged in 30% sucrose overnight and then transferred to a solution of 20% sucrose and OCT compound (50:50, v/v) at 4 °C overnight, for cryosectioning. The embedded embryos were sectioned at a thickness of 10 μm. For hematoxylin–eosin staining, the sections were sequentially soaked in the following solutions (and rinsed in running tap water between each staining step): 10% NDF solution for 10 s, hematoxylin solution for 2 min, ammonia solution for 10 s, eosin solution for 15 s, and 95% ethanol for 10 s. The sections were then soaked in 100% ethanol for 10 s three times, transferred to a 100% ethanol:xylene solution (v/v: 1/1) for 10 s three times, and rinsed with xylene for 10 s twice. For immunofluorescence staining, each section was placed on a glass slide, fixed with 4% paraformaldehyde for 10 min, and rinsed with PBS three times for 10 min each. Subsequently, the section was treated with 0.1% Triton X-100 in PBS and then soaked in a blocking solution (PBST containing 1% horse serum) at room temperature for 30 min. The sections were reacted with rabbit anti-HSP27 polyclonal antibodies (1:200, ab17938; Abcam, Cambridge, UK) at 4 °C overnight. After washing with PBS three times, the sections were treated with FITC-conjugated goat anti-rabbit IgG antibodies (1:200, AP132F, Chemicon/Merck Millipore, Billerica, MA, USA) at room temperature for 1 h. After extensive washing with PBS, the slides were treated with mouse Pan-Neuronal Marker antibodies (1:100, MAB2300, Chemicon/Merck Millipore, Billerica, MA, USA) at 4 °C overnight, and washed with PBS three times. The slides were then treated with Cy3-conjugated goat anti-mouse IgG antibodies (1:200, AP124C; Chemicon/Merck Millipore, Billerica, MA, USA) at room temperature for 1 h. After extensive washing, the slides were stained with DAPI (1:1,000, 71-03-00; KPL, Gaithersburg, MD, USA) for 10 min. The mouse embryos were visualized and digitized using the Zeiss Mirax Scan system (Carl Zeiss GmbH).

**Video legends**

**Video 1.** Morphological changes between HSP27-silenced and Luc-silenced PDMCs after IBMX induction

**Video 2.** Calcium influx and efflux in the induced neuron derived from HSP27-silenced PDMCs
